# Understanding changes in pulmonary function and functional status in breast cancer patients after systemic chemotherapy and radiotherapy: a prospective study

**DOI:** 10.1186/s12890-024-02890-5

**Published:** 2024-02-14

**Authors:** Alper Tuğral, Zeynep Arıbaş, Murat Akyol, Yeşim Bakar

**Affiliations:** 1 Faculty of Health Sciences, Department of Physiotherapy and Rehabilitation, Izmir Bakırçay University, Izmir, Turkey; 2 Faculty of Health Sciences, Department of Physiotherapy and Rehabilitation, Trakya University, Edirne, Turkey; 3 Faculty of Medicine, Department of Medical Oncology, Izmir Bakırçay University, Izmir, Turkey

**Keywords:** Breast cancer, Pulmonary function test, Chemotherapy, Radiotherapy, Six-minute walk test

## Abstract

**Background:**

Respiratory complications in breast cancer (BC) patients after chemotherapy (CT) and radiotherapy (RT) have been well acquainted and these complications should be investigated to prevent secondary problems and/or improve BC patients’ clinical outcomes. Therefore, this study aimed to assess the potential acute effect of systemic chemotherapy and radiotherapy on respiratory function and functional status of patients with breast cancer.

**Methods:**

A total of 25 BC patients who were candidates for systemic chemotherapy and radiotherapy were recruited after oncological examination and included in this study. Respiratory function and functional status were assessed with the Pulmonary Function Test (PFT) and the Six-Minute Walk Test (6MWT), respectively. Patients were assessed before CT (c0), after CT (c1), and after RT (r1).

**Results:**

25 BC patients were assessed in c0 and c1 while only 15 out of 25 patients (60%) were assessed in r1. The actual values of Forced vital capacity (FVC) (t = 2.338, *p* =.028), Forced expiratory volume in 1s (FEV1 (t = 2.708, *p* =.012), and the forced expiratory flow of between 25% and 75% of vital capacity (FEF25-75%) (t = 2.200, *p* =.038) were found significantly different after systemic CT. Inspiratory (MIP) and expiratory (MEP) muscle strength also did not show a significant change from c0 to c1. A significant effect of the type of surgery was found (Wilks’ lambda, F [1, 19] = 6.561, *p* =.019, ηp^2^ = 0.25) between c0 and c1 in actual FVC value. The main effect of time was found significant in FVC (F [2, 28] = 4.840, *p* =.016, ηp^2^ = 0.25) from c0 to r1. Pairwise comparisons with Bonferroni correction showed that there was a significant difference between c0 and r1 (*p* =.037).

**Discussion:**

The present study showed decreased FVC and FEV1 actual values and percent predicted rates from baseline to the completion of treatment. Since the interactional effect of the type of surgery was significant, we suggest that clinical and demographic factors such as age should be considered when interpreting the early changes in PFT. In addition, the significant linear trend of decreasing in some specific outcomes in respiratory function also highlighted the need for continuous monitoring of potential respiratory problems in patients with BC from baseline to the completion of chemotherapy and radiotherapy.

## Introduction

Breast cancer (BC) is the most frequent type of cancer seen in women globally with the incidence of BC as having nearly 13% [1]. However, the disease-free survival (DFS) rates of BC have also improved in recent decades. The reported rate of ten-year DFS is 90% [2].

The treatment of BC includes multimodal approaches such as surgery, chemotherapy (CT), radiotherapy (RT) as well and hormone therapy, therefore the potential experienceable symptoms of side effects of these multimodal treatments can remarkably vary [3].

Numerous experienceable symptoms can be discussed in survivorship such as breast cancer-related lymphedema, pain, upper extremity problems, chemotherapy-induced peripheral neuropathy, and so forth in BC survivors [3]. However, the potential impact of systemic chemotherapy and locoregional radiotherapy on the respiratory system of BC patients is one of the important points that should be discussed in detail during active systemic treatment of BC patients in order to provide them an optimal survivorship in the long term [4]. Paclitaxel, which is a frequently used chemotherapeutic agent in breast cancer was reported to be responsible for respiratory symptoms such as non-productive cough, wheezing, dyspnea, shortness of breath, etc. [4].. Ding et al. [5] also reported that dyspnea is the major symptom experienced by patients after systemic chemotherapy probably the latent effects of chemotherapeutic agents of respiratory musculature. In contrast, studies did not report any significant correlation and/or clinical significance between experienced symptoms and radiological images of the respiratory system [5–7]. On the other hand, locoregional radiotherapy, which is one of the most commonly used treatment modalities to reduce recurrence, especially in patients with breast-conserving surgery has also been reported to be responsible for respiratory symptoms due to the irradiation of thoracic structures [8]. Studies also reported decreased respiratory muscle strength (8) as well as significant changes in pulmonary function test (PFT) after locoregional radiotherapy [6, 8–10]. However, those studies also reported that no change in radiological images although significant decreases were observed in PFT. In addition, Briasoulis et al. [11] reported that the rate of interstitial pneumonia or pulmonary fibrosis associated with lung injury due to chemotherapy was 9%. Krengli et al. [10] also reported a rate of 4.9% as they had grade I pneumonitis which was resolved after medical treatment. However, the onset of potential respiratory problems due to radiotherapy may remarkably vary and the measurement times may eventually affect the relative volatility of the reported rates which ranged between 51 and 90% [12–15].

It is a well-known fact that chemotherapy itself may remarkably contribute to pulmonary toxicity with a variety of systemic effects. Yet, it was also reported that higher risk of radiation pneumonitis in patients with BC who underwent both chemotherapy and radiotherapy [5, 8–10]. However, there is a need for more studies in order to reflect the potential side effects of systemic treatment and their changes in respiratory symptoms in BC patients. PFT is a widely used instrument in clinical settings due to its safe operability and cost-effectiveness as well as its objective power to show early changes in the respiratory system. Not only for its useful application but also for some basic features such as Forced vital capacity (FVC) and forced expiratory volume in 1s (FEV1) which can be used to have an opinion about lung elasticity, size of airways, as well as resistance to flow through passages [16]. Since there might be problems associated with systemic chemotherapy and radiotherapy in terms of the deteriorated elasticity and increased resistance to the airflow of the airways, the PFT can be an optimal choice to detect potential early changes associated with respiratory problems. Therefore, we aimed to evaluate the potential acute changes in pulmonary function and functional status of BC patients, especially within the context of systemic treatment from pre-chemotherapy to the completion of radiotherapy via the pulmonary function test and the six-minute walk test (6MWT), respectively. Additionally, we also aimed to assess the impact of potential acute change in respiratory function on functional capacity as assessed via cardiorespiratory fitness.

## Method

### Study design

This study was planned as a prospective observational study and followed The Strengthening the Reporting of Observational Studies in Epidemiology (STROBE) guideline [17]. This study was held between February 2022 and January 2023. The study protocol was approved by Izmir Bakircay University Ethical Board of Clinical Studies with the 206/188. Patients were recruited in the outpatient clinic of the medical oncology unit at Bakircay University Cigli State and Training Hospital. This study was performed according to the 1964 Helsinki Declaration and its later amendments or comparable ethical standards.

### Patients

Patients who had been diagnosed with breast cancer and referred to medical oncology were screened and invited to participate according to the following inclusion and exclusion criteria: Being a volunteer to participate, having communication skills in the mother language, and having been selected for systemic chemotherapy were set as inclusion criteria. Being not a candidate for systemic chemotherapy, having mental and/or cognitive deficits, ongoing medical and/or surgical complications, and having neurological/orthopedical and/or severe respiratory dysfunctions were set as exclusion criteria. A signed informed consent was taken for each patient who was willing to participate in this study. Measurement periods were set as follows: Before chemotherapy (c0), after chemotherapy (c1, within fifteen days after the last CT cycle), and after radiotherapy (r1, within fifteen days after the last RT cycle).

### Assessments

#### Pulmonary function test

Pulmonary Function Test (PFT) was performed via a spirometer (Pony FX Spirometer; COSMED, Rome, Italy) in a standard sitting position. Actual and percent predicted rates of the following parameters were used and interpreted in the analysis according to the guidelines of the American Thoracic Society and European Respiratory Society: Forced vital capacity (FVC), forced expiratory volume in 1s (FEV1), peak expiratory flow (PEF), and FEF25-75%: The forced expiratory flow of between 25% and 75% of vital capacity. Respiratory muscle strength was also assessed with Maximum Inspiratory Pressure (MIP) and Maximum Expiratory Pressure (MEP) via a portable mouth apparatus (MicroRPM; Micro Medical London, United Kingdom) according to the aforementioned guidelines [18]. All procedures were performed by a physical therapist who had over twenty years of experience in respiratory rehabilitation (Z.A.).

### Functional status (6MWT)

Functional status of patients was assessed with the six-minute walk test (6MWT). This test is a cheap, objective, and safe option to have an opinion about the functional ability of different kinds of patients especially for ones with respiratory problems [19]. Briefly, a 30-m indoor corridor setting was established. Each patient was informed about the test and that they should walk as fast as possible for six minutes. Heart rate, SPO_2,_ perceived dyspnea, and fatigue were assessed in the before, after, and recovery phases of the test via a pulse oximeter (Nonin Medical, Model 2500, Plymouth, USA) and Modified Borg Scale, respectively. The total walked distance (TWD) was recorded as meters (m). The reliability of this test was reported to be 0.93 among cancer patients [20]. The 6MWT procedure was performed by only one researcher (A.T) in order to provide a standardization of this test.

### Statistical analysis

The data was presented as mean and standard deviation or number and percentages in continuous and categorical variables, respectively. The normality was controlled via skewness, kurtosis, and KS-SW normality tests. The paired data was assessed via paired t-test or repeated measures analysis of covariance (ANCOVA) especially when it was necessary to control some variables such as age or BMI between two paired data. A post hoc power analysis according to the FVC value showed that we achieved over 90% power by having an effect size of nearly 0.6 when taking into account partial eta squared (ηp^2^: 0.25) [21]. Therefore, we ceased to involve new BC patients to use time and resources effectively. Correlations were assessed via Pearson’s r or Spearman rho correlation coefficient. A *p*-value was accepted as significant below 0.05. Statistical analyses were performed via IBM SPSS v.20 (IBM Corp, Armonk, USA).

## Results

**Fig. 1:**
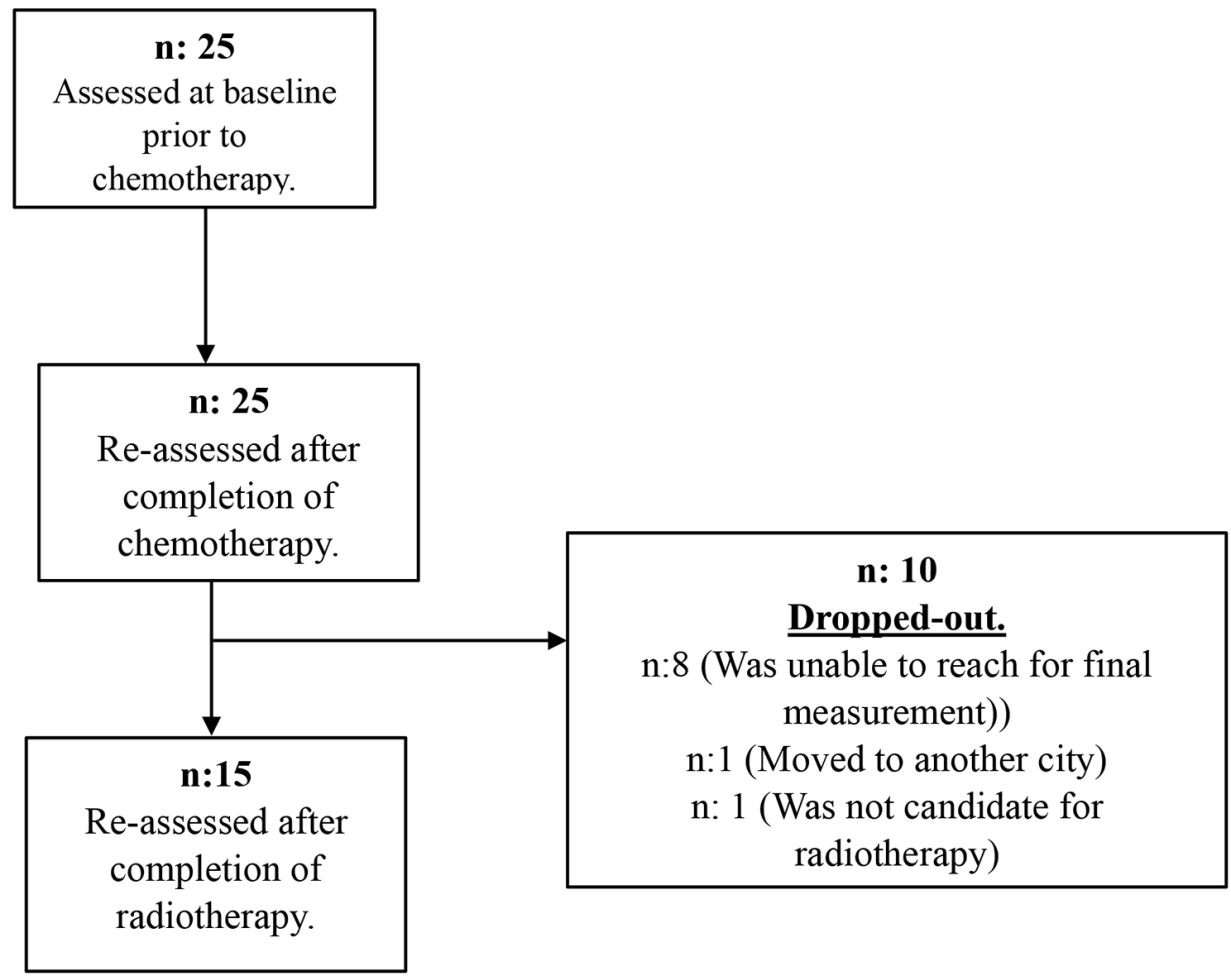
Flow chart of the study process

A total of 25 patients with breast cancer were included in this study. The mean age and body mass index (BMI) of patients were 48.86 ± 9.79 years and 27.59 ± 4.00 kg/m^2^, respectively. 15 out of 25 patients were assessed before and after CT as well as after RT. The details of participation and drop-out are shown in Fig. 1. 25 patients were assessed only before and after CT. Only 2 patients were undergoing neoadjuvant CT. 19 out of 25 patients had conservative breast surgery. 22 out of 25 patients underwent Anthracycline (Adriamycin + Cyclophosphamide) and Paclitaxel CT, while the rest of them underwent Docetaxel CT (Taxotere + Cyclophosphamide). The mean total dosage of RT was 53.75 ± 7.11 Gy for patients who were also re-assessed after RT (*n* = 15). The mean exposure of total dose and median cycle of CT was 1778.60 ± 665.39 mg and 14.08 ± 4.49, respectively. Patients who had conservative breast surgery (*n* = 6) had an extra boost for 5 cycles of RT (Mean: 10 Gy). The demographic and clinical characteristics of patients are shown in Table 1.

**Table 1:** Sociodemographic and Clinical Characteristics of Patients

*n* = 25	n (%)
**Type of CT protocol**	**ACT**
ACT	23(92)
NACT	2(8)
**Occupational Status**	
Active working	4(16)
Not working	21(84)
**Type of Chemotherapy Regimen**	
AC + PAXL	21(84)
TC	4(16)
**Type of breast surgery**	
Conservative	17(74)
MRM	6(26)
**Stage** ^*^	
T1	9(36)
T2	14(56)
T3	2(8)

**ACT**: Adjuvant Chemotherapy, **NACT**: Neo-Adjuvant Chemotherapy, **AC**: Adriamycin **+** Cyclophosphamide, **PAXL**: Paclitaxel, **TC**: Taxotere **+** Cyclophosphamide (Docetaxel), **MRM**: Modified radical mastectomy

^*^Staging was performed via histopathological staging for ACT patients or clinical staging for NACT patients

The actual values of FVC (t = 2.338, *p* =.028), FEV1 (t = 2.708, *p* =.012) and FEF25-75% (t = 2.200, *p* =.038) were found significantly different after systemic chemotherapy. No significant difference was obtained in actual values of FEV1/FVC and PEF. Percent predicted rates did not change significantly in all parameters of PFT before and after CT. Inspiratory (MIP) and expiratory (MEP) muscle strength also did not show a significant change from c0 to c1. Although a nearly 22 m decrease was observed in TWD at c1 compared to the baseline, this change was also not significant (t = 1.718, *p* =.102) in the total sample. Although we had a small number of patients who had MRM (*n* = 6), the mean difference of TWD was higher in patients with MRM compared to the patients who had conservative breast surgery (51.76 ± 90.92 m vs. 12.65 ± 41.10 m). Yet, no significant difference was obtained in within-group comparisons (t = 1.151, *p* =.270 for conservative group, t = 1.273, *p* =.272 for MRM). The mixed model also did not show a significant difference in the main effect of time (F [1, 17] = 0.692, *p* =.417, ηp^2^ = 0.03) on TWD when setting the type of surgery as a covariate. The interaction effect of *time x type of surgery* also did not show significance (F [1, 17] = 1.741, *p* =.205, ηp^2^ = 0.09).

When significant PFT results were analyzed according to the type of surgery (Conservative vs. MRM), no significant difference was found in actual FVC value in the main effect of time (Wilks’ lambda, F [1, 19] = 0.394, *p* =.538, ηp^2^ = 0.02) after setting the age and BMI as covariates as well as setting the type of surgery was between factor in GLM. However, a significant effect of the type of surgery was found (Wilks’ lambda, F [1, 19] = 6.561, *p* =.019, ηp^2^ = 0.25) between c0 and c1 in actual FVC value. The result was also significant with the same model as setting the FEV1 as the dependent variable (Wilks’ lambda, F [1, 19] = 5.012, *p* =.037, ηp^2^ = 0.20). Yet, in FEF25-75%, no significant difference was observed in the same model specifically the effect of the type of surgery (Wilks’ lambda, F [1, 19] = 0.100, *p* =.756, ηp^2^ = 0.05). The details of PFT results of patients before and after CT are shown in Table 2.

**Table 2:** Pulmonary function test results of patients before and after systemic chemotherapy

	Mean (SD)					
*n* = 25	cT0	cT1	Mean difference (SD)	95% CI	t	*p*
**FVC**	2.96 ± 0.63	2.83 ± 0.58	0.13 ± 0.28	0.015/0.25	2.338	**0.028**
**FEV1**	2.39 ± 0.40	2.28 ± 0.45	0.11 ± 0.20	0.02/0.19	2.708	**0.012**
**FEV/FVC**	81.76 ± 7.01	81.14 ± 6.23	0.62 ± 5.60	-1.69/2.92	0.550	0.587
**PEF**	4.48 ± 0.86	4.46 ± 1.24	0.0008 ± 1.03	− 0.41/0.44	0.042	0.967
**FEF_25–75**	2.50 ± 0.55	2.30 ± 0.58	0.19 ± 0.45	0.012/0.38	2.200	**0.038**
**FVC % (pred)**	87.44 ± 13.55	84.12 ± 14.45	3.32 ± 8.37	− 0.13 ± 6.78	1.982	0.059
**FEV1% (pred)**	88.12 ± 11.84	84.68 ± 14.71	3.44 ± 7.09	0.51/6.37	2.424	0.023
**FEV/FVC % (pred)**	101.12 ± 9.51	100.4 ± 8.14	0.72 ± 6.97	-2.15/3.59	0.517	0.610
**FEF_25–75% (pred)**	94.20 ± 25.77	86.60 ± 23.05	7.60 ± 19.15	− 0.30/15.50	1.984	0.059
**MIP**	68.86 ± 19.16	65.48 ± 18.27	3.38 ± 11.85	-2.01/8.77	1.307	0.206
**MIP % (pred)**	86.62 ± 24.11	83.67 ± 22.06	2.95 ± 15.25	-3.99/9.89	0.887	0.386
**MEP**	74.21 ± 20.61	74.26 ± 24.59	− 0.04 ± 16.62	-7.23/7.14	-0.013	0.990
**MEP % (pred)**	80.17 ± 20.45	84.65 ± 24.11	-4.48 ± 23.48	-14.63/5.67	-0.915	0.370

**FVC**: Forced vital capacity, **FEV1**: Forced expiratory volume in 1s, **PEF**: Peak expiratory flow, **FEF 25–75**: Forced expiratory flow of between 25% and 75% of vital capacity, **Pred**: Predicted, **MIP**: Maximal Inspiratory pressure, **MEP**: Maximal expiratory pressure, **SD**: Standard deviation, **cT0**: Before chemotherapy, **cT1**: After chemotherapy, *p* <.05., **t**: Paired samples t-test, **CI**: Confidence interval

15 out of 25 patients were also evaluated in post-RT (rT1) in addition to the c0 and c1. The main effect of time was found significant in FVC (F [2, 28] = 4.840, *p* =.016, ηp^2^ = 0.25). Pairwise comparisons with Bonferroni correction showed that there was a significant difference between c0 and r1 (*p* =.037). No significance was obtained between c1 and r1 as well as c0 and c1. Linear trend was also observed from pre-CT through post-RT (3.07–2.90 L, F [1, 14] = 8.277, *p* =.012, ηp^2^ = 0.37). The main effect of time was also significant in FEV1 (F [2, 28] = 7.815, *p* =.002, ηp^2^ = 0.36). Pairwise comparisons with Bonferroni correction showed that there was a significant difference between c0 and r1 (*p* =.004). No significance was obtained between c1 and r1 as well as c0 and c1. Yet, a marginal *p*-value (0.051) was obtained between c0 and c1. A significant linear trend was also observed from pre-CT through post-RT (2.48–2.33 L, F [1, 14] = 15.707, *p* =.001, ηp^2^ = 0.52). In addition, there were also significant main effects of time in predicted percent rates of FVC and FEV1. The pairwise comparisons showed that there were significant differences between c0 and c1 as well as c0 and r1 in predicted rates of FVC. However, in predicted rates of FEV1, pairwise comparisons showed a significant difference between c0 and r1. The main effect of time showed no significant difference in FEV/FVC, PEF, FEF25-75% as well as MIP and MEP values from c0 through r1. The interaction effect of *time x type of surgery* not only showed no significant difference in MIP and MEP values from c0 to r1 but also in FVC and FEV1 values in the same measurement times. The details of the PFT values of patients who were assessed before and after CT and RT (c0, c1, and r1) are shown in Table 3.

**Table 3:** Pulmonary function test results of patients before and after systemic chemotherapy and radiotherapy

	Mean (SD)					
*n* = 15	cT0	cT1	rT1	F	*p*	ηp^2^
**FVC**	3.07 ±.55^a^	2.93 ± 0.49	2.90 ±.54^a^	4.840	**0.016**	0.25
**FEV1**	2.48 ±.38^a^	2.37 ± 0.39	2.33 ±.41^a^	7.815	**0.002**	0.36
**FEV/FVC**	81.28 ± 0.4.50	81.07 ± 4.82	80.56 ± 4.60	0.188	0.811	0.013
**PEF**	4.71 ± 0.86	4.85 ± 1.18	4.50 ± 0.80	1.447	0.25	0.09
**FEF_25–75**	2.59 ± 0.53	2.43 ± 0.55	2.39 ± 0.57	2.770	0.087	0.16
**FVC % (pred)**	88.93 ± 10.50^**a,b**^	85.20 ± 10.03^**a**^	84.33 ± 10.42^**b**^	4.145	**0.027**	0.23
**FEV1% (pred)**	89.00 ± 9.41^**a**^	85.40 ± 9.83	84.07 ± 10.40^**a**^	6.372	**0.006**	0.31
**FEV/FVC % (pred)**	100.06 ± 5.97	99.87 ± 5.68	99.40 ± 5.46	0.105	0.885	0.007
**FEF_25–75% (pred)**	93.06 ± 21.28	86.80 ± 16.63	85.80 ± 17.16	2.344	0.123	0.14
**MIP**	68.57 ± 17.67	64.69 ± 18.70	66.73 ± 18.44	0.669	0.506	0.05
**MIP % (pred)**	84.50 ± 22.63	82.30 ± 20.86	83.20 ± 22.34	0.343	0.68	0.028
**MEP**	71.35 ± 18.39	69.86 ± 24.92	71.47 ± 23.81	0.217	0.768	0.016
**MEP % (pred)**	75.71 ± 18.95	80.64 ± 25.39	80.33 ± 19.75	0.463	0.596	0.034

**FVC**: Forced vital capacity, **FEV1**: Forced expiratory volume in 1s, **PEF**: Peak expiratory flow, **FEF 25–75**: Forced expiratory flow of between 25% and 75% of vital capacity, **Pred**: Predicted, **MIP**: Maximal Inspiratory pressure, **MEP**: Maximal expiratory pressure, **SD**: Standard deviation, *p* <.05, **ηp**^**2**^: Partial eta-squared, **F**: Repeated Measures ANOVA, **cT0**: Before chemotherapy, **cT1**: After chemotherapy, **rT1**: After radiotherapy

^a^Significantly different between two measurement times according to the Bonferroni Post-hoc test

^b^Significantly different between two measurement times according to the Bonferroni Post-hoc test

There were also significant correlations that were noteworthy to be noticed. Age was significantly correlated with both baseline and post-CT actual values of FVC (c0: *r*=-.594, *p* =.002, c1: *r*=-.549, *p* =.004), FEV1 (c0: *r*=-.572, *p* =.003, c1: *r*=-.531, *p* =.006), and FEV/FVC (c0: *r* =.429, *p* =.032, c1: *r* =.160, *p* =.445), except for the value of FEV/FVC obtained in c1. Age was also significantly correlated with the baseline TWD (r=. -576, *p* =.003). TWD was also found significantly correlated with FVC (*r* =.505, *p* =.010) and FEV1 (*r* =.525, *p* =.007) in the baseline and post-CT values of FVC (*r* =.481, *p* =.015) and FEV1 (*r* =.452, *p* =.023). However, post-CT (c1) TWD values of patients did not show any significant correlation with the PFT results. BMI was significantly correlated with FEV/FVC value both in c0 (*r* =.427, *p* =.033) and c1 (*r* =.438, *p* =.028). No significant correlations were obtained in PEF and FEF25-75% values.

## Discussion

The present study showed significant changes in actual, and percent predicted rates of specific PFT values such as FVC and FEV1 before and after systemic chemotherapy and these changes were linearly and significantly decreased after the completion of RT. Since this study relatively aimed to assess the potential changes in the near trajectory of systemic and locoregional treatment, it is evident that even some changes can occur during and after just completion of systemic treatment. Although a decrease in inspiratory muscle strength was observed before and after systemic chemotherapy, this change did not reach significance either in MIP or MEP values. On the other hand, clinical characteristics such as type of breast surgery had a significant effect on actual FVC and FEV1 results before and after chemotherapy. In addition, the effect of age has also highlighted itself by showing significant correlations with specific PFT values not only in baseline but also in the post-CT. Though a decrease was observed in TWD, it did not reach the significance. Yet, it should be noted that baseline TWD might be used to predict pulmonary function due to the significant correlation between the TWD and post-CT FVC and FEV1 values. Multivariate analyses and post-hoc tests showed that FVC and FEV1 actual values and predicted percent rates differed significantly between pre-CT and post-RT. In this manner, our results seem parallel with the literature findings [7]. Therefore, it is possible to draw a conclusion that these changes should be carefully monitored and interpreted due to the multifactorial aspect of the potentially hazardous effects of systemic chemotherapy and radiotherapy as well as the reversible nature of some parameters within a longer term. Although the changes in functional status and respiratory muscle strength were negligible, significant decreases in FVC and FEV1 might be noteworthy to take action on potential problems in survivorship among breast cancer patients as soon as possible.

Systemic chemotherapy has been widely used as a primary treatment option for breast cancer due to its proven efficacy and contribution to survival. However, due to its systemic application, adverse effects can be widely seen during chemotherapy in patients with breast cancer. These side effects can sometimes show themselves as systemic due to the potential impact on the heart, kidneys, and lungs in a prolonged period [22]. It was stated that BC patients might experience dyspnea after chemotherapy, and it was also thought that it is associated with injury of respiratory muscles and peripheral nerves due to chemotherapy agents [23]. On the other hand, it should also be noted that patients with cancer can face a wide variety of symptoms including fatigue, which is one of the most common symptoms of patients with cancer especially during chemotherapy [24]. Studies also highlighted that the potential reason for increased dyspnea might be the result of skeletal and respiratory muscle weaknesses due to cytotoxic CT agents [25]. Paclitaxel, which is known as the most frequently used chemotherapeutic agent in solid tumors such as breast cancer, might cause respiratory symptoms like non-productive cough, shortness of breath, and tightness in the chest [26, 27]. Yoshioko et al. [28] also reported that Nab-Paclitaxel is responsible for drug-induced lung injury at the rate of nearly 13% of patients who underwent Paclitaxel. Yet, the authors also stated that many patients were asymptomatic. In parallel with this, Ding et al. [5] reported no change in chest X-rays, however, significantly increased ventilatory volume and perceived dyspnea as well as a decrease in diffusion capacity for carbon monoxide (DLCO) were also reported in their study. In this regard, PFT was reported to be an important clinical outcome to detect, interpret, and guide changes in potential pulmonary toxicity by presenting specific test results in earlier settings [6]. In our study, we found significant changes in FVC and FEV1 before and after chemotherapy as well as after RT. Although our results were significant, these changes did not reach clinical significance due to the mean decrease was quite below 20%. In addition, we did not find any significant changes in the rest of the parameters of PFT. Our insignificant results seem to parallel with the findings of Ding et al. [5] and Dimopoulou et al. [7]. However, it should also be taken into account that some differences between our study and theirs that all patients had undergone neoadjuvant CT in Ding’s study, whereas patients in Dimopoulou’s study had various types of malignancies such as bladder, ovarian, endometrial, and cervical except for breast cancer. On the other hand, the type of surgery was found to have a significant effect on FVC and FEV1 values before and after chemotherapy. Although the great majority of our sample underwent breast-conserving surgery, this was an expected result due to patients with MRM might experience more difficulty with PFT because limited chest wall mobility [8] and altered postural kinematics [29] were well-known consequences after MRM. Anthracyclines are reported to be less likely to cause pulmonary toxicity on their own, yet when used in combination with other drugs the risk of toxicity may increase [30]. Since there was no significant change in other parameters of PFT before and after CT, this result can be attributed to the drug regimen used in our study in which the great majority of patients’ CT was combined with Anthracycline and paclitaxel. Practically, FEV1/FVC value can be used to detect obstructive ventilatory defect as having below 0.70 as an indicator of obstructive pattern. In the baseline, there was only one patient who had an FEV1/FEV value of 0.70, and who had also a BMI over 35 kg/m^2^. This result can also be supported by the significant correlation between BMI and FEV1/FVC. However, the mean FEV1/FVC ratio of our patients was above 0.80 in all measurement points. However, predicted percents were also higher than 80%, therefore we can conclude that there was no significant change in respiratory function in these acute settings. It may also be reasonable to attribute these changes to our measurement times which might not have been sensitive to detect potential changes related to the systemic treatments. Since radiation-induced lung injury and related symptoms might arise nearly one to four months after completion of radiotherapy [10]. Nonetheless, it was also reported that FEV1 was a significant predictor of the long-term diffusion capacity (DLCO) [7], which was reported to be the most reliable parameter for detecting changes in respiratory function [5, 6]. Although DLCO was reported to be the most sensitive parameter, conflicting results were also reported [8]. Yet, it should also be considered that changes in FEV1 and FVC over time also depend on socio-clinical demographics as well as baseline lung function [31]. Studies also reported that changes in PFT should be thoroughly interpreted due to their relatively large variability [9]. The significant correlations obtained in our study between the parameters of PFT and age underline the importance of the management of potential respiratory function especially in older breast cancer patients. Similarly, we found no significant main effect of time when age and BMI were set as covariates.

The six-minute walk test (6MWT) has been widely used in the cancer population due to its simple operability and ease of interpretation to predict survival and treatment outcomes. Since it mimics daily life activities, it is frequently used to have an opinion about functional status from the perspective of cardiorespiratory fitness [32, 33]. In this regard, we also assessed the 6MWT of patients before and after chemotherapy to detect potential changes in cardiorespiratory fitness associated with systemic chemotherapy. Although a nearly 22 m decrease was observed after systemic chemotherapy in the total group, this result did not reach significance. It was also reported that breast cancer survivors had nearly 35 m less TWD than their healthy counterparts [34]. Despite the greater decrease observed in TWD in patients with MRM surgery, it was also not significant compared to the patients with breast-conserving surgery. Similarly, we recently showed that there was no significant difference in TWD in patients with surgery and without throughout the systemic chemotherapy after controlling for age and BMI [35]. Similarly, to our previous results, age was also found to have a significant correlation with the baseline TWD in our present study. In addition, significant correlations between the baseline TWD and FVC as well as FEV1 results also highlighted the importance of the potential effect of respiratory function on functional status. Notably, the significant correlation between those with baseline TWD and post-CT PFT values might be considerable in order to be used to predict pulmonary function after systemic chemotherapy. By performing this simple field test, not only to predict pulmonary function but also to assess physical function can be achieved in cancer survivors. Lewandowska et al. [36] stated that decreased physical function was the key factor for the deteriorated quality of life in cancer patients.

Post-operative radiotherapy is one of the main treatment modalities in patients with breast cancer especially for who underwent breast-conserving surgery to mitigate the risk of locoregional recurrence and improve survival. Yet, irradiation of other vital structures such as lung tissue may cause respiratory toxicity and other respiratory symptoms which may last for nearly months after completion of RT [8, 37]. Locoregional RT was identified as a risk factor for radiation-induced pneumonitis and fibrosis [38]. A recent review also highlighted the need for early management of the potential risk of radiation-induced damage to the lungs and heart [39]. Therefore, potential changes in PFT might be the reflections of these symptoms. Karlsen et al. [40] reported that patients with MRM were at nearly 2.5-fold higher risk of showing clinical radiation pneumonitis and fibrosis within three months of completion of RT. Cilla et al. [41] reported that postmastectomy RT was associated with an increased risk of cardiac and respiratory complications such as pericarditis and radiation-induced fibrosis. Yet, the interactional effect of the type of surgery and the main effect of time on specific respiratory parameters such as FVC and FEV1 did not show significance in our study from baseline to completion of radiotherapy. Alsaeed et al. [42] reported a significant decrease in FVC and FEV1 values of patients with MRM after ninety days of completion of RT, however, they also indicated that no patient was symptomatic in their pilot study. However, we found significant changes from pre-CT through the completion of RT in FVC and FEV1 results. Our results seem in parallel with other studies [8]. In addition, pairwise comparisons also revealed that a significant decrease was observed between baseline and post-RT values. A linear trend was also found significant. This significant trend and percent decrease in these parameters were also evident and comparable with our study (4.5% vs. 3.3%) [9] in FEV1, FVC parameters same as in our study. However, no significant change in respiratory muscle strength could be attributed to the measurement times of our study. Since we assessed patients within fifteen days after completion of RT, potential changes related to the probable respiratory deterioration could not be assessed due to the inadequate time of cumulative effects of RT. However, studies also reported that patients who underwent weekly paclitaxel in addition to irradiation treatment showed significantly shorter time associated with pulmonary symptoms [22]. In contrast, Suesada et al. [8] reported a decrease in respiratory muscle strength in their patients, yet the authors assessed respiratory function after three months of completion of RT. Likewise, the same authors also reported no significant change in DLCO, unlike other studies. This result can be interpreted as even after three months after completion of RT, the changes that might reflect themselves as clinically significant may not be in the clinical situation. Besides, studies also underline the timeline of late reactions at least six months after completion of RT which can also be characterized with or without symptoms [43]. Borst et al. [44] reported a decrease in DLCO and FEV1 parameters up to three years after RT. However, it should also be considered when interpreting the changes in PFT, many studies performed PFT on patients with lung cancer which have already a close relationship to disease progression except for patients with breast cancer. In addition, they also reported that supraclavicular lymph node irradiation (SCLN) and chest wall tightness might have contributed to their results, yet it may not be valid for the sample due to the type of surgery and SCLN which were mostly breast-conserving surgery and there was no SCLN, respectively. Also, we did not find a significant interaction effect of the type of surgery from pre-CT to post-RT in FVC and FEV1. We did not analyze the potential effect of the history of smoking on PFT values due to there was no patient who reported herself as a current smoker. Yet, studies also revealed that smoking history was not a significant contributor to the PFT values [6, 9, 10].

This study has also some strengths and limitations that should be acknowledged. We had a nearly 40% loss to follow-up after the completion of RT, therefore we had to analyze the data from pre-CT to post-RT only in 15 patients. Since there was a limited time and busy clinical schedule, we also were not able to assess the 6MWT of patients after RT, therefore we could only analyze the TWD before and after CT. We did not assess the DLCO values, unlike other studies. Although no patient reported any signs of respiratory symptoms such as ongoing cough, we did not use any valid measure of respiratory symptoms. Furthermore, the timing of the last measurement (r1) may not have detected changes as it may have been too early for potential tissue damage. Finally, a relatively small sample size can account for the limitations of this study. Yet, we assess patients in the trajectory of their systemic treatment and analyze by controlling other variables in a relatively cost-effective manner can account for the strength of this study. We suggest that further studies may include perceived symptom assessment scales associated with daily functional living and respiratory function.

## Conclusion

The present study showed a significant decrease in actual values of FVC and FEV1 of patients not only before and after chemotherapy, but also after completion of radiotherapy with a significant linear trend. Although many studies reported that these decreases are reversible within a nearly one-year period after completion of systemic treatment [9, 10], these outcomes can be supported by other objective measures such as 6MWT to detect earlier changes and take precautions as soon as possible. Even though significant decreases were not meaningful in the context of clinical implication, they might be interpreted as early signs of potential deterioration of respiratory function. Moreover, patients with higher age and/or having mastectomy should be thoroughly monitored due to they can be more vulnerable to suffering from respiratory complications associated with systemic treatment.

## Data Availability

The data can be available from the corresponding author upon reasonable request and with permission of Bakircay University Ethical Board of Clinical Studies.

## Abbreviations

BC: Breast cancer
CT: Chemotherapy
RT: Radiotherapy
PFT: Pulmonary function test
6MWT: Six-minute walk test
c0: Before chemotherapy
c1: After chemotherapy
r1: After radiotherapy
FVC: Forced vital capacity
FEV1: Forced expiratory volume in 1 s
FEF25-75%: The forced expiratory flow of between 25% and 75% of vital capacity
MIP: Maximum inspiratory pressure
MEP: Maximum expiratory pressure
ηp2: Partial eta-squared
DFS: Disease-free survival
STROBE: The Strengthening the Reporting of Observational Studies in Epidemiology
PEF: Peak expiratory flow
TWD: Total walked distance
m: Meters
ANCOVA: Analysis of covariance
KS-SW: Kolmogorov-Smirnov, Shapiro-Wilk
BMI: Body mass index
Gy: Gray
MRM: Modified Radical mastectomy
DLCO: Diffusion capacity for carbon monoxide
